# GC-MS Analysis and Various In Vitro and In Vivo Pharmacological Potential of *Habenaria plantaginea* Lindl.

**DOI:** 10.1155/2022/7921408

**Published:** 2022-03-31

**Authors:** Mater H. Mahnashi, Yahya S. Alqahtani, Bandar A. Alyami, Ali O. Alqarni, Mohammad Ahmed Alshrahili, Mahrous A. Abou-Salim, Mohammed N. Alqahtani, Sadaf Mushtaq, Abdul Sadiq, Muhammad Saeed Jan

**Affiliations:** ^1^Department of Pharmaceutical Chemistry, College of Pharmacy, Najran University, Najran, Saudi Arabia; ^2^Armed Forces Hospital Jazan, Jazan, Saudi Arabia; ^3^Al-Azhar University, Faculty of Pharmacy, Pharmaceutical Organic Chemistry, Assiut 71524, Egypt; ^4^Ahad Rufaidah General Hospital, Abha-Ahad Rufaidah, Jazan, Saudi Arabia; ^5^ELMS College, Springfield Street, Chicopee, MA, USA; ^6^Department of Pharmacy, Faculty of Biological Sciences, University of Malakand, Chakdara 18000, Dir (L), KP, Pakistan; ^7^Department of Pharmacy, University of Swabi, Anbar 23561, Swabi, KPK, Pakistan

## Abstract

*Background*. The current study aims to give a scientific origin for employing *Habenaria plantaginea* Lindl. as a potential candidate against nociception, inflammation, and pyrexia. The pharmacological studies were performed on crude extract and subfractions. In the gas chromatography-mass spectroscopy analysis, a total of 21 compounds were identified. The plant samples were displayed for in vitro anti-inflammatory potentials. The observed IC_50_ for chloroform against cyclooxygenase-2 and 5-lipoxygenase enzymes was 33.81 and 26.74 *μ*g/mL, respectively. The in vivo activities were prerequisites with the acute toxicity studies. In carrageenan-induced inflammation, the chloroform fraction exhibited 46.15% inhibition similar to that of standard drug diclofenac sodium 47.15%. Likewise, in the acetic acid-induced writhing test, the ethyl acetate fraction displayed 71.42% activity, which was dose-dependent as that of standard drug. In Brewer's yeast-induced antipyretic activity, a significant decrease in rectal volume was observed after 30, 60, and 90 minutes. Moreover, the results of this study indicated that the chloroform and ethyl acetate fractions inhibited nociception, inflammation, and pyrexia dose dependently. Likewise, mechanistic insights indicated that naloxone antagonized the antinociceptive effect of chloroform and ethyl acetate fractions, thereby signifying the involvement of opioidergic mechanisms respectively. These results suggest that these molecules present in this plant have synergistically beneficial potential for the cure and management of analgesia, inflammation, and pyrexia.

## 1. Introduction

New drug from a natural source with a desirable profile is tremendously an inspiring task in the field of clinical research [1]. Nociceptive pain induces activation of sensory nerve fibers when the stimulus overshoots the noxious intensity [2]. Chemical, thermal, and mechanical stimuli are the various common types of nociceptive pain which might further be alienated into deep, visceral, superficial somatic, and somatic pain [3]. The visceral part of the organ is extremely sensitive to inflammation, ischemia, and stretch but is not sensitive to the other stimuli that induce pain in other parts of the body [4]. Deep somatic pain initiates in blood vessels, muscles, tendons, bones, and ligaments after the stimulations of nociceptors in these organs. This type of pain is dull and poorly localized. Muscle sprain and breaking bones are the most common examples of deep somatic pain [5]. When the nociceptors in superficial and skin tissues activate, they induce superficial pain, which is very sharp. Minor burns and minor wounds are the most common examples of superficial pain [6]. Pain is a sign to propel a person from deleterious conditions to protect a damaged part of the body after healing. After removing the toxic stimuli, most of the pain sensation disappears but in some cases, pain perception remains for a long time after removing the damaging stimuli. Inflammation is a complicated protective response involving immune cells, blood vessels, and molecular mediators of the body to harmful stimuli, damaged cells, or invading microbes [6, 7]. The main role of inflammation is to eliminate of necrotic cells, damaged tissues, and injured cells and to start the tissue repairing process of the body [8]. The immune response of the body is not specific of a body to irritation, infection, and injury, which may be acute or chronic [9].

The clinical hallmarks of inflammations in Latin are rubor (redness), calor (warmth), tumor (swelling), and dolor (pain). The characteristics of inflammation were first described by the Roman physician Aulus Cornelius Celsus (Aurelius) [10]. The body response against the invading microbes or any other foreign substances is the innate immune system that is specific for specific microorganisms that enter into the body [11]. Inflammation is divided into two types: acute and chronic inflammation.

Presently, mild-to-moderate pain, inflammation, and pyrexia are managed with NSAIDs, but there are serious limitations of these therapies [12]. For example, there is well-documented evidence that continuous use of NSAIDs displays toxicity such as GIT ulceration, bleeding, perforation, cardiovascular disorders, and analgesic nephropathy. Hence, across the globe, extensive search is in progress in the area of medical management of pain and inflammation to find out new remedies [13]. This new drug discovery acts as alternative drugs to traditional analgesics including NSAIDs and narcotics.

*Hebanaria genus* is associated with the Orchidaceae family, which may consist of about 850 genera and 35000 species [14, 15]. Orchids were employed as the basis of conventional remedy for a very long time to cure various disorders such as arthritis, syphilis, stomach problem, acidity, tumor, jaundice, boils, inflammations, piles, hepatitis, malaria, blood dysentery, pyrexia, sexually transmitted ailments, tuberculosis, cholera, wounds, eczema, vermifuge, and diarrhea [16–18]. *Bulbophyllum neilgherrense*, a traditional therapeutic plant, has lately been assessed as a mediator of inflammation and analgesia [19]. The anti-inflammatory properties of orchids from South Africa have also been studied [20]. No scientific evaluation has been reported for the investigation of pharmacological features of *H. plantaginea*. In our previous study, we have also explored *H. digitata*, which belongs to the same family for its anti-inflammatory and analgesic potential [21].

We have deliberated this research work to explore the pyrexia, analgesia, and anti-inflammatory properties of *H. plantaginea* based on ethnomedicinal exploitation and previous literature assessment. Furthermore, we have indomitable phytochemicals via GC-MS methods. Evaluation of these unexplored plants can potentially lead to the generation of new molecules for drug development and better medicine.

## 2. Materials and Methods

### 2.1. Chemicals and Drugs

For anti-inflammatory in vitro assay, the enzyme COX-2 (Catalog no. C0858) and 5-LOX (Catalog no. 437996) were acquired from the Sigma-Aldrich GmbH, USA. The substrate arachidonic acid (CAT no. 150384), linoleic acid (CAS no. 60-33-3), and their cofactor solution materials TMPD (CAS no. 637-01-4), hematin (CAS no. 15489-90-4), and glutathione (CAS no. 70-18-8) were purchased from Sigma-Aldrich, Germany. Carrageenan (CAS no. 9064-67-7), naloxone, and AA (CAS No: 506-32-1) were also acquired from Sigma-Aldrich, Germany. Buffer solution containing KH_2_PO_4_ and K_2_HPO_4_ and solvents employed were of pure class. Celecoxib and Montelukast was purchased from Pfizer pharmaceutical and Libra (Pvt.) Limited. Tramadol and Diclofenac were purchased from Alliance Pharmaceuticals, Pakistan.

### 2.2. Plant Material, Collection, and Extraction

The *H. plantaginea* plant was collected from the Dir (*L*) KPK, Pakistan, in mid of April and then was identified via Prof. Muhammad Ilyas, Department of Botany, University of Swabi, Swabi KPK, Pakistan. The sample of the said plant was kept and recorded at herbarium having voucher number H.UOS.20-2. The aerial pieces of the plant (15 kg) were soaked with uncontaminated water and were sheltered desiccated for 21 days. The sheltered desiccated pieces of plants were firstly incised into minor pieces and then via grinder changed into coarse fine particles (7.5 kg). The pulverized substance was then macerated in the 26 L methanol (80%) for 21 days. Afterward, the entire substance was filtered using the muslin cloth and consequently by the Whatman filter paper. Then, the filtrate was transferred to a rotary evaporator (40°C) for further extraction [2, 22]. The last gloomy green color hard methanolic extort of *H. plantaginea* was attained weighing 650 g.

### 2.3. Fractionation

Methanol extort was decanted quietly in the separating funnel having a closed stopper. The Hp. Cr was mixed with the same amount of 500 ml hexane and water. The separating funnel was shacked vigorously for proper mixing of all the ingredients and then reserved at the correct position via a stand to prepare two separate layers: the *n*-hexane and water layer. The layer of *n*-hexane was alienated. A similar process was repeated two times through 500 ml of hexane. The organic layers which were separated three times were then mixed and concentrated in decreased pressure via rotary evaporator having a temperature of 40°C. The obtained concentrated weight of Hex. was 27.6 g. Similarly, the identical protocol was applied with the further solvents via raising the polarization of solvents. Following solvent fractions acquired was basically of ethyl acetate, chloroform, and Bt., having weights of 30, 42, and 94 g correspondingly. In last, the aqueous stratum was concentrated with a weight of 140 g [23].

### 2.4. GC-MS Analysis (Phytochemistry)

The tandem gas chromatography/mass spectrometry procedure of crude extract was executed by Agilent USB:393752 gas chromatograph (Agilent Technologies, Palo Alto, CA, USA) having an HHP-5MS 5% phenyl methyl siloxane tubular-column (30.0 m × 0.25 mm × 0.25 *μ*m film thickness: Restek, Bellefonte, PA, USA) prepared through the Agilent HP; 5973 mass selective detector having the effective mode of electron (Ionization energy; 70 eV) performance in the parallel investigational ambiance as exemplification, intended for gas chromatography [10, 24].

### 2.5. In Vitro Pharmacological Activities

#### 2.5.1. COX-2 Activity

The in vitro COX-2 scavenging activity was conceded through the previous explained normal protocol [25]. The COX-2 solution of the enzyme was equipped to have 300 U ml^−1^ concentrations. For enzyme activation, the enzyme solution (10 *μ*l) was placed on ice for 5 minutes. Likewise, total of 50 *μ*l cofactor mixture with 0.9 mM glutathione, 1 mM hematin, and 0.24 mM TMPD within 0.1 M Tris HCl buffer (pH 8.0) was added to a mixture of the solution of enzyme. Consequently, plant samples (20 *μ*l) including different concentrations (1000–31.25 *μ*g ml^−1^) along with 60 *μ*l of the solution of the enzyme were placed at room temperature for five minutes. Similarly, 30 mM AA (20 *μ*l) was added for starting the reaction. After that, the solution mixture was incubated for 5 min. After the incubation, the absorbance of the solution mixture was recorded at 570 nm using UV-visible spectrophotometer. The COX-2 enzyme inhibition was indomitable from per unit time of absorbance value. The IC_50_ values were resolute by plotting the reticence of enzyme beside different test fraction concentrations. Celecoxib was employed as a positive control (standard drug).

#### 2.5.2. 5-LOX Assay

The inhibitory potential of 5-LOX on the *H. plantaginea* various fractions was carried out as per the previously reported procedure. Firstly, various dilutions were made ranging from 31.25 to 1000 *μ*g/mL. Afterward, the 5-LOX enzyme having 10,000 U ml^−1^ solutions was prepared. Linoleic acid (80 mM) was used as a substrate in this assay. Likewise, phosphate buffer (50 mM) was ready with 6.3 pH. The various fractions of the plant samples were mixed in phosphate buffer solution and lipoxygenase enzyme (250 *μ*l each) was mixed with it and incubated for 5 minutes at normal room temperature. Then, 0.6 mM of substrate solution (1000 *μ*l) was added with that of the solution containing enzyme and shaken; after that, absorbance was deliberated at 234 nm. The experimental procedures were carried out thrice. In our activity, the standard drug employed was zileuton [26]. The % inhibition was calculated by the following equation:

(1) $$\begin{array}{c} \text{Percentage Inhibition}=\frac{\text{Control Abs}.-\text{Sample Abs}.\;}{\text{Control Abs}.\;}\times 100. \end{array}$$

### 2.6. In Vivo Pharmacological Activities

#### 2.6.1. Animals

Albino mice of either sex were used in all experimental work and all experiments were conducted in the time range from 8.00 am to 5.00 pm. Food and water were available to all animals. The light-and-dark cycle was conserved with a temperature of 22 ± 2°C and which has an exhaust fan facility. At the end of all experimental processes, animals were sacrificed by scheduled 1 method and appropriately disposed of according to ethical guidelines of the institution. The experimental albino mice were employed as per authorization of the ethical board having letter number: UOS. Ph. 40121 Department of Pharmacy, University of Swabi, Pakistan.

#### 2.6.2. Acute Toxicity

The acute toxicity was carried out on investigational albino mice. All animals were alienated into different sets having test and control groups. Every group has 5 tested animals. The *H. plantaginea* tested samples were administered per oral at various doses (25 to 2000 mg kg^−1^) according to body weight. For the dose preparation, Tween-80 was employed. After administration of various doses, the experimental mice were pragmatic for up to 3 days for any minor allergic reactions and abnormal behavior [27].

#### 2.6.3. Antinociceptive Activity

We used two standard models for the evaluation of antinociceptive activity in mice: acetic acid-induced writhes and hot plate models [28, 29].

*(1) Acetic Acid-Induced Writhes*. Animals were deprived of food and water for 2 hours before starting the experimental procedure. Animals were divided into various groups. The acetic acid at a dose of 10 ml/kg (1%) was administered intraperitoneally into the animal, which leads to constrictions of the abdomen. This constriction was counted for 20 minutes [23]. Diclofenac sodium at a dose of 50 mg/kg was taken as a reference standard. Crude extract and their subsequent fractions were given at doses of 100 mg/kg, while 0.9% sodium chloride was taken as a control group. The standard drug, test drug, and normal saline were administered intraperitoneally to different groups of animals 30 minutes before the administration of acetic acid.

*(2) Hot Plate Test*. All the animals were habituated to the laboratory environment at least 2 hours before experimental procedures. Analgesiometer was used for the evaluation of analgesic activity on hot plate [30]. The hot plate temperatures were kept at 54.0 ± 0.1°C. Animals were subjected to pretest and all those animals that show latency time less than 30 seconds were selected. The selected animals were then arranged into various groups like normal saline group, standard group, and other potent fractions of the tested samples. The normal saline, standard drug, and various fractions of the test sample were administered intraperitoneally. The latency time of animals on the hot plates at intervals of 30, 60, and 90 minutes was calculated.

*(3) Effect of Naloxone on Antinociceptive Activity of Various Fractions on Hot Plate Model Experimental Protocol*. For the evaluation of the mechanism of the *H. plantaginea*, naloxone and tramadol were given to the animals. The animals were divided into ten groups, each group containing six animals (*n* = 6). All the animals were acclimatized to the laboratory environment 1 hour before the start of experimental work. In this study, albino mice of either sex were used through experiments and are exposed to the hot plate having a temperature of 54.0 ± 0.1°C. All the animals were subjected to pretest. All those animals were selected which shows latency time less than 30 seconds and the other animals were rejected to circumvent tissue damage [29]. After completion of the pretest, tramadol at a dose of 5 mg/kg was injected intraperitoneally and the latency time was recorded at 30, 60, and 90 minutes. In the antagonistic activity, naloxone at a dose of 1 mg/kg was administered subcutaneously 10 minutes before tramadol administrations [31].

#### 2.6.4. Anti-Inflammatory Activity

*(1) Carrageenan-Induced Inflammation*. Carrageenan-induced paw edema was used for evaluation of anti-inflammatory activity [32]. All the animals of either sex were arranged into five groups each having six animals. All the animals were fasted overnight but have free excess to water. Diclofenac sodium (50 mg/kg) as a standard drug, normal saline as a control group, and various fractions at a dose of 100 mg/kg were administered 30 minutes before administration of 0.05 ml of 1% carrageenan into the subplantar area of the paw. A high-sensitivity instrument digital plethysmometer was used for measuring a small volume change in hid paw developed in the form of edema after injection of carrageenan. Reading was taken on a plethysmometer before and after carrageenan administrations up to 5 hours at an interval of 1 hour.

#### 2.6.5. Antipyretic Activity

**Figure 1:**
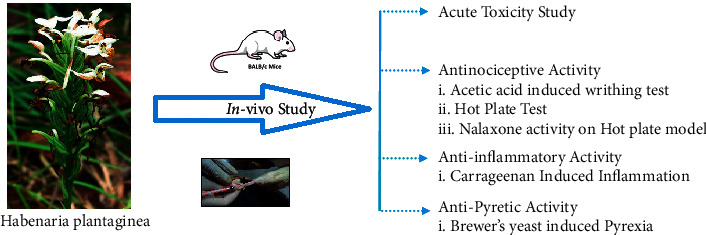
Schematic diagram of the in vivo experimental animal design.

*(1) Brewer's Yeast-Induced Pyrexia*. For the evaluation of antipyretic study, Brewer's yeast model was used in which 20% Brewer's yeast was injected subcutaneously leading to hyperpyrexia [33]. All the animals were fasted overnight having free excess to water only and are arranged into various groups, each group containing 6 animals. With the help of a digital thermometer, rectal temperatures were recorded after 24-hour administration of Brewer's yeast. All those animals were selected which shows the rise in temperature up to 0.3–0.5°C. Group 1 received normal saline at a dose of 10 ml/kg, group 2 received standard drug at a dose of 150 mg/kg, and groups 3, 4, and 5 received test drug at a dose of 100 mg/kg body weight intraperitoneally. Rectal temperature was recorded after administration of all groups at an interval of 30 minutes, 60 minutes, and 90 minutes. Lubricants such as olive oil were used for insertion into the animal rectum and were maintained for 30 seconds for temperature record (Figure 1).

#### 2.6.6. Analysis of Data

The results of all the experimental works were demonstrated as mean ± SEM of all the groups having six animals. The IC_50_ values were calculated through SPSS software. One-way ANOVA statistical tools were used for the assessment and differences of various means, which follow two-way ANOVA followed by Bonferroni's posttest by using GraphPad Prism version 5. During statistical analysis, the value of *P* that is less than 0.05 was considered to be statistically significant.

## 3. Results

### 3.1. GC-MS Analysis

**Figure 2:**
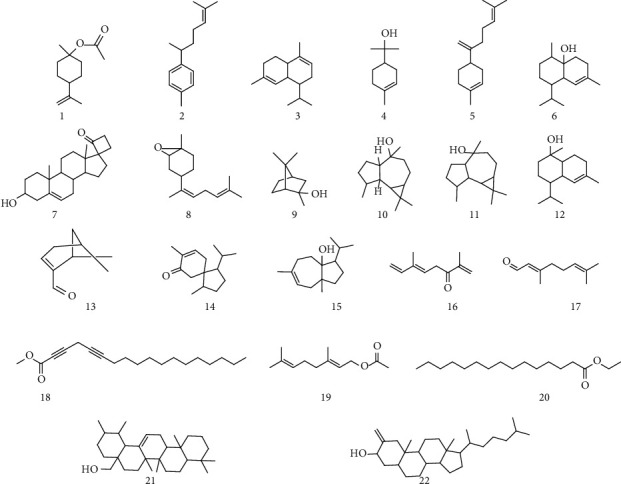
Identified compounds' structures in *Habenaria plantaginea*.

The GC-MS analysis of Hp. Cr was executed and recognized twenty-two (22) molecules in it. The structure of all the known molecules is exhibited in Figure 2. GC-MS identification depends on the corresponding of spectral peak, the fragmentation pattern of its peak and mass through the identified library is established inside the apparatus. For that reason, occasionally it is probably because of the fact that basic two molecules will have the same fragmentation pattern and mass spectra, but it is not an ordinary case. Furthermore, all the details about the new molecule present inside of plant were not installed in GC-MS library, so there is the probability of not identifying those new molecules. Full details of GC-MS analysis are summarized in Table 1. The compounds 2, 4, 5 were previously reported for their anti-inflammatory potential while compound 6 was reported as antihemorrhagic, analgesic, diuretic, antipyretic, and insecticide activities.

### 3.2. *In Vitro* Pharmacological Activates

#### 3.2.1. Cox-2 Assay

In this assay, various fractions like Hp. Chf and Hp. EtAc exhibited excellent COX-2 inhibition as shown in Table 2. Hp. Chf showed that the highest COX-2 inhibitory potential observed was 77.40 ± 0.25,72.41 ± 0.30, 65.79 ± 1.28, 61.32 ± 0.68, and 56.49 ± 0.73%; activity was monitored for the fraction Hp. Chf at concentration of 1000, 500, 250, 125, and 62.5 *μ*g mL^−1^, respectively, with IC_50_ value of 33.81 *μ*g mL^−1^. Likewise, Hp. EtAc exhibited second highest inhibitory potential observed: 76.38 ± 0.76, 69.37 ± 0.52, 62.90 ± 1.16, 54.48 ± 0.54, and 45.56 ± 0.69 with IC_50_ value 87.56 *μ*g mL^−1^, respectively. Celecoxib demonstrated 84.51 ± 0.30, 77.84 ± 0.27, 73.50 ± 2.26, 65.74 ± 0.16, and 61.56 ± 0.28 with IC_50_ value 23.30 *μ*g mL^−1^ respectively. All the other fractions displayed good-to-moderate activity in this assay.

#### 3.2.2. 5-LOX Assay

In this assay, Hp. Chf exhibited highest activity against with 81.73 ± 0.37, 75.27 ± 1.37, 69.62 ± 0.11, 63.81 ± 0.51, and 59.08 ± 0.12 percent inhibition at concentration of 1000, 500, 250, 125, and 62.5 *μ*g mL^−1^, respectively. The IC_50_ value measured from the dose-response curve was 26.74 *μ*g mL^−1^. Similarly, Hp. EtAc exhibited 80.47 ± 0.70, 73.57 ± 0.43, 65.12 ± 0.94, 57.76 ± 1.09, and 49.38 ± 0.50 with IC_50_ value 67.51 *μ*g mL^−1^, respectively. The standard drug linoleic acid displayed 87.66 ± 0.45, 81.64 ± 0.42, 76.01 ± 1.61, 70.46 ± 0.32, and 64.50 ± 0.02 inhibition at concentration ranging from 1000 to 62.5 *μ*g mL^−1^, respectively, attaining the IC_50_ value of 17.47 *μ*g mL^−1^ as shown in Table 2.

### 3.3. In Vivo Pharmacological Activities

#### 3.3.1. Acute Toxicity Result

In the acute toxicity studies, no mortality and no changes in behavior were noticed in the investigational mice. Based on the observation in the acute toxicity result, a dose of 2000 mg/kg was measured as the safest dose. The information of the doses specified to mice is presented in Table 3.

#### 3.3.2. Evaluation of Antinociceptive Activity

**Figure 3:**
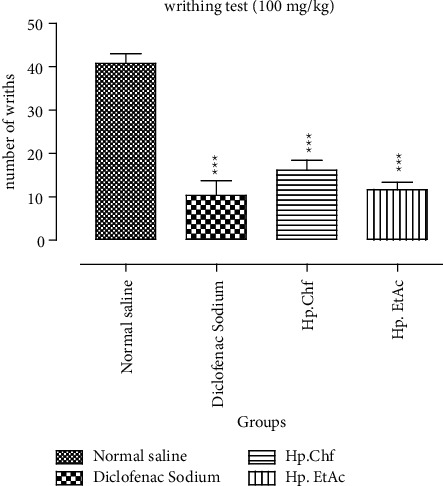
Analgesic activities through acetic acid-induced writhing potential in mice. The data were expressed as means ± SEM (*n* = 6). Data were analyzed via two-way ANOVA followed by Bonferroni's posttest. ^*∗∗∗*^*P* < 0.001. ^###^Comparison of standard drug to the normal saline group.

*(1) Acetic Acid-Induced Writhing Activity*. A significant decrease in the number of writhing was observed in animals treated with standard drug (50 mg/kg I/P) as compared to the normal saline control group. The number of writhes exhibited by the standard group is 10.33 with 74.70% of inhibition, while the Hp. Chf and Hp. EtAc have also reversed the effect of acetic acid and displayed number of writhes 16.17 ± 0.10 and 11.67 ± 0.60 (*P* < 0.001) with 60.39 ± 0.42, and 71.42 ± 0.55% of inhibition, respectively, at a dose of 100 mg/kg I/P (Figure 3).

*(2) Evaluation of Antinociceptive Activity on Hot Plate Model after 30 Minutes*. A significant increase in the latency time was observed in animals treated with standard drug (tramadol 5 mg/kg I/P) as compared to the vehicle control group. The latency time after 30 minutes observed in the positive control group was 17.23 with 47.59 ± 0.71% analgesic activity at 100 mg/kg body weight. The Hp. Chf fraction of the plant has reversed the latency time 14.33 ± 0.44 (*P* < 0.01) with 36.99 ± 0.94% inhibition of thermal stimuli significantly at the dose of 100 mg/kg I/P. The Hp. EtAc also reversed the effect significantly causing 29.62 ± 0.84 (*P* < 0.05) % inhibitions at the dose of 100 mg/kg (Table 4).

*(3) Evaluation of Antinociceptive Activity on Hot Plate Model Result after 60 Minutes*. A significant increase in the latency time was observed in animals treated with standard drug (tramadol 5 mg/kg I/P) as compared to the vehicle control group after 60 minutes (*P* < 0.001). The fraction Hp. Chf has reversed the analgesic potential causing 44.16 ± 0.70% inhibition (*P* < 0.01) significantly at the dose of 100 mg/kg I/P. The tested fraction Hp. EtAc having latency time in 14 seconds, which has also reversed the good effect of thermal stimuli with 35.50 ± 0.50% inhibition (*P* < 0.05) significantly at the dose of 100 mg/kg (Table 4).

*(4) Evaluation of Antinociceptive Activity on Hot Plate Model Result after 90 Minutes.* A significant increase in the latency time was observed in animals treated with standard drug (tramadol 5 mg/kg I/P) as compared to the vehicle control group with latency time 13.27 (*P* < 0.001) causing 34.69 ± 0.56 of % inhibition. The Hp. Chf fractions reversed the pain effect significantly with latency time 11.25 (*P* < 0.01) causing percent analgesic inhibitions 22.93 ± 0.60 at the dose of 100 mg/kg I/P. The Hp. EtAc fraction displayed a latency time of 8.8 causing a very low effect as contrasted to the negative control at the same dose. Results were shown in Table 4.

*(5) Mechanistic Antinociceptive Activity of Various Fractions on Hot Plate Model.* It depicts the tramadol antinociceptive activity, which shows that standard drug morphine (5 mg/kg) possesses a significant result, 34.10 ± 0.20 (*P* < 0.001) % inhibition; the tested fraction Hp. Chf at a dose of 100 mg/kg causes 58.23 ± 0.44 (*P* < 0.001) % inhibition while Hp. EtAc exhibited significant result 43.39 ± 0.52 (*P* < 0.001).  Table 5 represents the effects of naloxone after 30, 60, and 90 minutes in hot plate test. The data were expressed as means ± SEM (*n* = 6).

#### 3.3.3. Evaluation of Anti-Inflammatory Activity Test Results

**Figure 4:**
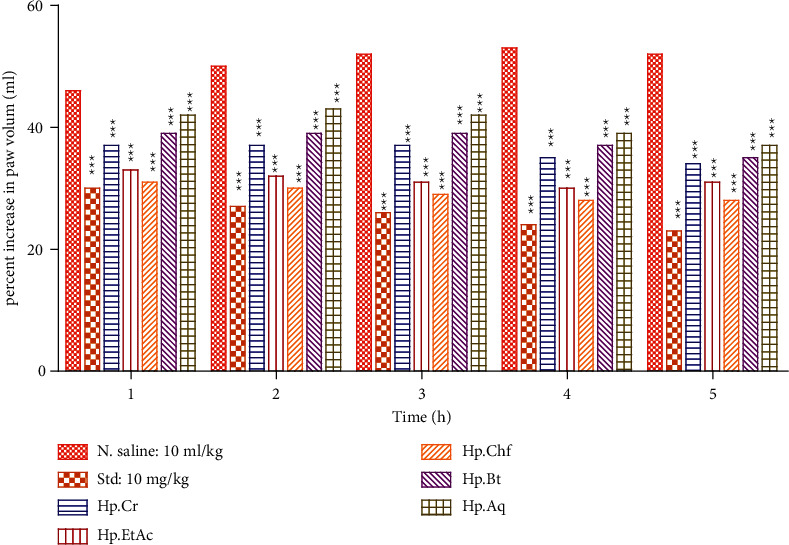
Carrageenan-induced anti-inflammatory potential in mice. The data were expressed as means ± SEM (*n* = 6). Data were analyzed via two-way ANOVA followed by Bonferroni's posttest. ^*∗∗∗*^*P* < 0.001.

A significant decrease in paw volume was observed in animals treated with standard drug (diclofenac sodium 10 mg/kg I/P) as compared to the vehicle control group. The percent increase in paw volume in the positive control group is decreased from the first 1^st^ to 5^th^ h with 30.01 ± 0.10 to 23.20 ± 0.40 (*P* < 0.001) causing 34.76 ± 0.56 to 55.38 ± 0.80% edema volume inhibition from the 1st to 5th hours, respectively. The Hp. Chf displayed 32.61 ± 0.88 percent inhibition at the 1st h and till significant 46.15 ± 0.10 at the 5th h. The next most potent activity was shown by Hp. EtAc at the 4th h (43.40 ± 0.42) after carrageenan induction (Figure 4).

#### 3.3.4. Evaluation of Brewer's Yeast-Induced Antipyretic Activity Result

**Figure 5:**
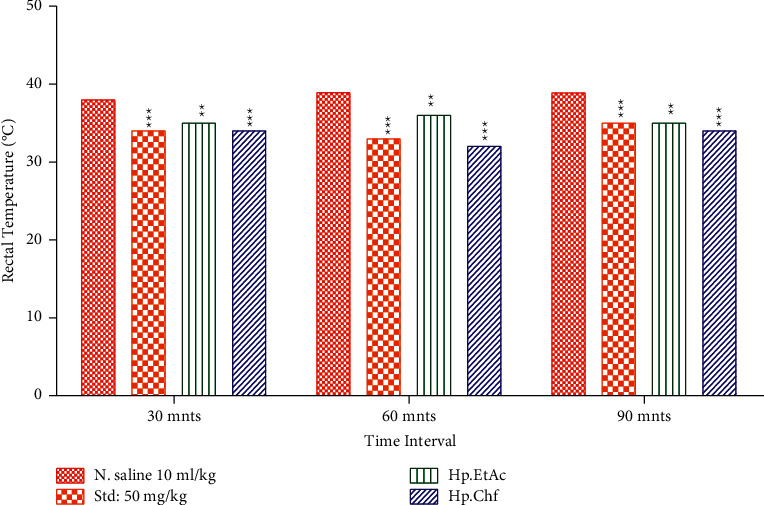
Anti-inflammatory activities through Brewer's yeast-induced pyrexia in mice. The data were expressed as means ± SEM (*n* = 6). Data were analyzed via two-way ANOVA followed by Bonferroni's posttest. ^*∗∗∗*^*P* < 0.001.

The subcutaneous injection of yeast suspension noticeably elevated the rectal temperature after administration. A significant decrease in rectal volume was observed after 30, 60, and 90 minutes with 34.20 ± 0.42, 33.02 ± 0.10, and 35.50 ± 0.52°C (*P* < 0.001) in animals treated with standard drug (50 mg/kg I/P) as compared to the vehicle control group. The tested fraction Hp. Chf also reduced the rectal temperature significantly at 30, 60, and 90 minutes causing 34.10 ± 0.56, 32.88 ± 0.66, and 34.10 ± 0.20°C (*P* < 0.001) at 100 mg/kg I/p. The other fraction like Hp. EtAc also revealed good reduction in rectal volume after 60 minutes, 36.20 ± 0.40°C (*P* < 0.01) (Figure 5).

## 4. Discussion

Currently, several investigational drugs have been developed by scientists to control pain and inflammation but still the therapy is not completely satisfactory [34, 35]. Due to a considerable increase in the field of medicine, disease and disorder treatment with new inventions increase tremendously. The evolutions of synthetic compounds and alternate medicine are searching out in which the possibilities of adverse effects should be minimized as compared to existing drugs. For the treatment and management of pain, inflammation, and pyrexia, the use of NSAIDs leads to severe abnormalities and complications which causes cardiac and kidney abnormality, gastric and intestinal bleeding, and so on (Wolfe et al., 1999) [27]. The human COX enzyme is a dimer of COX-1 and COX-2 subunits and catalyzes the oxidation of AA to generate PGG_2_ followed by reduction into PGH_2_ [36]. The prostaglandins PGG_2_ and PGH_2_ are the precursors of signaling molecules in various diseases including inflammation, cardiovascular problems, and cancer [37]. The catalytic domain of each subunit of the dimer comprises cyclooxygenase and peroxidase active sites on either side of the heme prosthetic group [38]. The nonselective NSAIDs target the COX-1 and COX-2 enzymes to block the formation of inflammatory signaling precursors leading to acute inflammation, cancer, and cardiovascular diseases [39]. For example, aspirin acetylates Ser-530 on the cyclooxygenase active site and inactivates the enzyme by interfering with the binding of AA to Ser-530 in each of the two subunits [40].

For the evaluation of antinociceptive activity acetic acid-induced writhing model, pain in the peripheral origin is induced through the injection of irritant chemicals, such as acetic acid [41]. In the activity of abdominal constrictions, which are induced by acetic acid, the synthesis of prostaglandins plays a key role [42] through the action of the essential enzymes such as cyclooxygenase-II, which cause an increase in pain perception at sensory nerve endings. Perception of pain through COX pathway and activation of the sensory pathways in the peritoneum of mice encourage abdominal constrictions and a viscerosomatic reflex was observed in response to acetic acid (algesiogenics) [43]. At the terminal of the abdominal peritoneum sensory afferents, neurons possess adrenergic receptors like *α*-adrenoceptors, *β*-adrenoceptors, and some opioid receptors. The generations of pain impulses become depressed with the activation of these receptors by the appropriate agonists but in the mice peritoneum, an interaction was found between opioid receptors and *α*-adrenoceptors [44, 45]. The central and peripheral processes are involved in abdominal constrictions [46].

The inflammatory process along with neurons of the dorsal horn is activated in the later phase of nociception [47]. It was suggested that in hot plate test determination of central pain mechanisms the nociceptive responses are integrated supraspinally [48].

In Figure 1, the study of antinociceptive activity on mice was evaluated through acetic acid induced writhing model, which shows a significant decrease in the number of writhes in animals treated with standard drug being 10.33 with 74.70% of inhibition at a dose of 50 mg/kg I/P. The potent fractions of *H. plantaginea* like Hp. Chf and Hp. EtAc at the dose of 100 mg/kg body weight I/p show a significant effect with the number of writhes: 16.17 ± 0.10 and 11.67 ± 0.60 causing 60.39 ± 0.42 and 71.42 ± 0.55% of inhibition, respectively (*P* < 0.001), in acetic acid-induced analgesia. Hot plat test has been performed to distinguish whether the various fractions have a central or peripheral antinociceptive effect. Standard drug shows a significant 47.59 ± 0.71 %inhibition (*P* < 0.001) resulting in prolonging the latency time at various intervals while in tested fractions Hp. Chf exhibited a significant result causing 36.99 ± 0.94 and 44.16 ± 0.70 (*P* < 0.01) after 30 and 60 minutes at a dose of 100 mg/kg I/P in hot plate evaluation test. The tramadol antagonistic nociceptive activity was evaluated by utilizing the hot plate test [49]. The antinociceptive effect of tramadol (5 mg/kg) was reversed significantly by opioid antagonist naloxone (1 mg/kg) causing 34.10 ± 0.20 (*P* < 0.01), and the Hp. EtAc and Hp. Chf fractions have shown a significant result: 43.39 ± 0.52 (*P* < 0.01) and 29.76 ± 0.66 (*P* < 0.01) percent inhibition after 30 minutes at a dose of 100 mg/kg, respectively.

For the evaluation of antipyretic activity, fungal pyrogens present in Brewer's yeast induce pyrexia in rodents. Recommended guidelines for the evaluation of antipyretic activities show that Brewer's yeast should be administered subcutaneously [50]. Upon administration of Brewer's yeast, several inflammatory mediators such as transcription factors, cytokines like IL-6 and TNF-, and enzymes involved in the synthesis of PGE_2_ are released [51]. The retardations of these mediators are accountable for the antipyretic effects (Rawlins, 1973). The cause of pyrexia induced with the administration of Brewer's yeast may be due to the production of prostaglandins [52]. Upon administration of various reactions of the *H. plantaginea* at high doses such as 100 mg/kg on the base of body weight I/P and standard drug paracetamol the rectal temperature of mice were significantly at 30, 60, and 90 minutes with 34.20 ± 0.42, 33.02 ± 0.10, and 35.50 ± 0.52°C (*P* < 0.001) reduced, which shows that the tested fraction possesses antipyretic activity. The possible mechanism of the Hp. Chf (34.10 ± 0.56 at 30 minutes) fraction and standard drug paracetamol may be the inhibition of PGs, which shows its antipyretic activities.

The most common model used for the study of inflammation is carrageenan-induced paw edema [53]. The most important parameter of inflammation is the formation of edema, which is considered for the evaluation of anti-inflammatory activity of selected compounds [54]. PGE_2_ and Bradykinin release at the site of inflammation induced carrageenan [55, 56]. A biphasic response was investigated with the injection of carrageenan in the paw of the mice in which multiple mediators are released which leads inflammation (Cuzzocrea et al., 1999). This study shows that Hp. Chf fraction of *H. plantaginea* significantly displayed 32.61 ± 0.88 percent inhibition at the 1st h and till significant 46.15 ± 0.10 at the 5th h. The next potent activity was shown by Hp. EtAc at the 4th h (43.40 ± 0.42) after carrageenan induction. The results demonstrate that the both fractions affect the early and late phases of inflammation.

During the pain and inflammatory progression, the release of free radicals like ROS that cause the proinflammatory cytokines (IL1*β*, TNF-*α*, and IL-6), production of cell lyses, and COX and LOX expression are responsible for various diseases [57, 58]. The inhibitory result of the various fractions of *H. plantaginea* against COX-2 and 5-LOX enzyme was assessed that were extensively employed to conclude the anti-inflammatory capacity of the fractions [59] and is generally employed for the evaluation of analgesic and anti-inflammatory potential of various fractions. Based on the result attained from the current study, the COX-2 and 5-LOX scavenging potential of Hp. Chf (IC_50_ 33.81 and 26.74 *μ*g/mL) exhibited comparable and dose-dependent results to that of standard drug celecoxib and montelukast (IC_50_ 23.20 and 17.47 *μ*g/mL) [60]. The in vitro analgesic and anti-inflammatory capacity of various fractions contribute to strengthening the anti-inflammatory, antipyretic, and analgesic potentials.

This study possesses significant importance in the development of new natural product research, which permits us to predict that the said plant has new drugs that contain antinociceptive, antipyretic, and anti-inflammatory properties. The natural products are free from common side effects possessed by traditional NSAIDs, which minimize economic burden and increase patient compliance. The current study possesses a base for the development of new drugs, which require detailed pharmacological study on different animal models.

## 5. Conclusions

Based on current results, it might be clear that *H. plantaginea* plant has anti-inflammatory potential and also possesses analgesic and antipyretic compounds. Further, *H. plantaginea* may be a good candidate for complementary and alternative therapy. This might be free from common side effects passed by traditional NSAIDs. This study also gives investigational and scientific justification for the ethnomedicinal use of *H. plantaginea* plant as analgesic, anti-inflammatory, and antipyretic.

## Figures and Tables

**Table 1:** GC-MS details of identified compounds.

S. no.	Chemical name	Common name/synonym	Formula
1	1-Methyl-4-(prop-1-en-2yl)cyclohexyl acetate	Beta-terpinyl acetate	C_12_H_20_O_2_
2	1-Methyl-4-(6-methyl hept-5-en-2-yl)benzene	Alpha-curcumene	C_15_H_22_
3	1-Isopropyl-4,7-dimethyl-1,2,4a,5,6,8a-hexahydronaphthalene	Not identified	C_15_H_24_
4	2-(4-Methylcyclohex-3-enyl)propan-2-0l	Alpha-terpineol	C_10_H_18_O
5	1-Methyl-4-(6-methylhepta-1,5-dien-2-yl)cyclohex-1-ene	*β*-Bisabolene	C_15_H_24_
6	1-Isopropyl-4,7-dimethyl-1,2,3,4,4a,5,6,8a-octahydronaphthalen-4a-ol	Cadina-1(6),4-diene	C_15_H_26_O
7	Spiro[androst-5-ene-17,1′cyclobutan]-2′one,3-htdroxy-	Not identified	C_22_H_32_O_2_
8	(Z)-1-Methyl-4-(6-methylhepta-2,5-dien-2-yl)-7-oxa-bicyclo[4.1.0]heptane	Not identified	C_15_H_24_O
9	2,7,7-Trimethylbicyclo[2.2.1]heptan-2-ol	Isoborneol	C_10_H_18_O
10	1,1,4,7-Tetramethyl-decahydro-1*H*-cyclopropa[e]azulen-4-0l	Viridiflorol	C_15_H_26_O
11	1,1,4,7-Tetramethyl-decahydro-1*H*-cyclopropa[e]azulen-4-0l	Globulol	C_15_H_26_O
12	4-Isopropyl-1,6-dimethyl-1,2,3,4,4a,7,8,8a-octahydronaphthalen-1-ol	*δ*-Cadinol	C_15_H_26_O
13	4,6-Dimetylcyclohex-1-enecarbaldehyde	Not identified	C_9_H_14_O
14	Spiro [4.5]dec-6-en-8-0ne, 1,7-dimethyl-4-(1-methylethyl)-	Acorenone 1	C_15_H_24_O
15	(Z)-3-isoropyl-6,8 a-dimethyl-1,2,3,3a,4,5,8,8a-octahydroazulen-3a-ol	Bullnesol	C_15_H_26_O
16	(E)-3,7-dim ethylocta-1,5,7-trien-3-one	Hotrienol	C_10_H_14_O
17	(E)-3,7-dimethylocta-2,6-dienal	Not identified	C_10_H_16_O
18	Methyl octadeca-2,5-diynoate	Methyl 2,5-octadecadiynoate	C_19_H_30_O_2_
19	(E)-3,7-dim ethylocta-2,6-dienyl acetate	1-Octanol	C_12_H_20_O_2_
20	Ethyl pentadecanoate	*n*-Pentadecanoic acid ethyl ester	C_17_H_34_O_2_
21	(1,2,6a,6b,9,9,12a-heptamethyl-1,2,3,4,4a,5,6,6a,6b,7,8,8a,9,10,11,12,12a,12b,13,14b-icosahydropicen-4a yl)methanol	Not identified	C_30_H_50_O
22	10,13-Dimethyl-2-methylene-17-(6-methylheptan-2-yl)-hexadecahydro-1H-cyclopenta[a]phenanthren-3-ol	Not identified	C_28_H_48_O

**Table 2:** Results of in vitro cyclooxygenase and lipoxygenase inhibitory activity.

Name	Concentration	COX-2% inhibition (mean ± SEM)	COX-2 IC_50_ (*μ*g/ml)	5-LOX % inhibition (mean ± SEM)	5-LOX IC_50_ (*μ*g/ml)
Hp. Chf	1000	77.40 ± 0.25^*∗∗∗*^	33.81	81.73 ± 0.37^*∗∗∗*^	26.74
	500	72.41 ± 0.30^*∗∗*^		75.27 ± 1.37^*∗∗∗*^	
	250	65.79 ± 1.28^*∗∗∗*^		69.62 ± 0.11^*∗∗∗*^	
	125	61.32 ± 0.68^*∗*^		63.81 ± 0.51^*∗∗∗*^	
	62.5	56.49 ± 0.73^*∗∗*^		59.08 ± 0.12^*∗∗∗*^	
Hp. EtAc	1000	76.38 ± 0.76^*∗∗∗*^	87.56	80.47 ± 0.70^*∗∗∗*^	67.51
	500	69.37 ± 0.52^*∗∗∗*^		73.57 ± 0.43^*∗∗∗*^	
	250	62.90 ± 1.16^*∗∗∗*^		65.12 ± 0.94^*∗∗∗*^	
	125	54.48 ± 0.54^*∗∗∗*^		57.76 ± 1.09^*∗∗∗*^	
	62.5	45.56 ± 0.69^*∗∗∗*^		49.38 ± 0.50^*∗∗∗*^	
Hp. Cr	1000	65.94 ± 0.71^*∗∗∗*^	200	71.50 ± 0.56^*∗∗∗*^	106.99
	500	58.28 ± 0.54^*∗∗∗*^		65.40 ± 0.55^*∗∗∗*^	
	250	52.65 ± 0.91^*∗∗∗*^		59.36 ± 0.57^*∗∗∗*^	
	125	45.30 ± 0.55^*∗∗∗*^		51.30 ± 0.52^*∗∗∗*^	
	62.5	37.63 ± 0.98^*∗∗∗*^		44.37 ± 0.58^*∗∗∗*^	
Hp. Hex	1000	64.55 ± 0.51^*∗∗∗*^	217.93	66.42 ± 0.46^*∗∗∗*^	171.05
	500	57.55 ± 0.67^*∗∗∗*^		60.53 ± 0.41^*∗∗∗*^	
	250	51.40 ± 0.44^*∗∗∗*^		52.68 ± 0.64^*∗∗∗*^	
	125	45.67 ± 0.55^*∗∗∗*^		47.46 ± 0.47^*∗∗∗*^	
	62.5	37.33 ± 0.62^*∗∗*^		40.51 ± 0.62^*∗∗∗*^	
Hp. Bt	1000	60.35 ± 0.51^*∗∗∗*^	438.39	63.45 ± 0.59^*∗∗∗*^	328.34
	500	51.27 ± 0.58^*∗∗∗*^		55.49 ± 0.60^*∗∗∗*^	
	250	43.41 ± 0.55^*∗∗∗*^		46.23 ± 0.44^*∗∗∗*^	
	125	34.40 ± 0.76^*∗*^		37.50 ± 0.61^*∗∗*^	
	62.5	27.24 ± 0.80^*∗*^		31.47 ± 0.46^*∗*^	
Hp. Aq	1000	68.83 ± 1.07^*∗∗∗*^	141.2	72.37 ± 0.54^*∗∗∗*^	132.27
	500	61.39 ± 0.60^*∗∗∗*^		64.00 ± 0.20^*∗∗∗*^	
	250	56.58 ± 0.56^*∗∗∗*^		57.15 ± 0.91^*∗∗∗*^	
	125	49.29 ± 0.43^*∗∗∗*^		51.15 ± 0.61^*∗∗∗*^	
	62.5	41.37 ± 0.58^*∗∗∗*^		40.40 ± 0.68^*∗∗∗*^	
Celecoxib	1000	84.51 ± 0.30	23.20	—	—
	500	77.84 ± 0.27			
	250	73.50 ± 2.26			
	125	65.74 ± 0.16			
	62.5	61.56 ± 0.28			
Montelukast	1000	—	—	87.66 ± 0.45	17.47
	500			81.64 ± 0.42	
	250			76.01 ± 1.61	
	125			70.46 ± 0.32	
	62.5			64.50 ± 0.02	

The values are presented as mean ± SEM (*n* = 5). The asterisk shows that the significance levels in comparison with that of the negative control. Data were analyzed via two-way ANOVA followed by Bonferroni's posttest. ^*∗*^*P* < 0.05, ^*∗∗*^*P* < 0.01, and ^*∗∗∗*^*P* < 0.001.

**Table 3:** Group of animals and drug quantities are given for acute toxicity studies with various fractions of *H. plantaginea*.

Groups	Animals	Conc. (*μ*g/mL)
1	5	25
2	5	50
3	5	100
4	5	200
5	5	300
6	5	400
7	5	500
8	5	1000
9	5	2000

**Table 4:** Antinociceptive activity of *H. plantaginea* assessed using the hot plate test.

Treatment	Dose (mg/kg)	Latency time in seconds (mean ± SEM)		
		After 30 min	After 60 min	After 90 min
Normal saline	10 ml/kg	9.03 ± 0.40	10.05 ± 0.30	8.67 ± 0.65
Standard	5 mg/kg	17.23 ± 0.33^*∗∗∗*^	19.56 ± 0.52^*∗∗∗*^	13.27 ± 0.25^*∗∗∗*^
Hp. Chf	100 mg/kg	14.33 ± 0.44^*∗∗*^	16.17 ± 0.42^*∗∗*^	11.25 ± 0.52^*∗∗*^
Hp. EtAc	100 mg/kg	12.83 ± 0.52^*∗*^	14.00 ± 0.10^*∗∗∗*^	8.83 ± 0.80^*∗*^

The data represent analgesic activities through hot plate test in mice. The data were expressed as means ± SEM (*n* = 6) analyzed via two-way ANOVA followed by Bonferroni's posttest. ^*∗*^*P* < 0.05; ^*∗∗*^*P* < 0.01; ^*∗∗∗*^*P* < 0.001; and ns: not significant.

**Table 5:** Results of analgesic activity following hot plate model and opioid receptors evaluation study.

Samples	Dose (mg/kg)	Reaction time on hot plate		
		30 min	60 min	90 min
Normal saline	10 ml/kg	10.12 ± 0.42	10.12 ± 0.57	10.12 ± 0.33
Normal saline + NLX	10 ml/kg + 1	10.14 ± 0.71	10.14 ± 0.47	10.14 ± 0.60
Morphine	5	21.64 ± 0.59	23.64 ± 0.70	19.64 ± 0.60
Morphine + NLX	5 + 1	14.26 ± 0.94	16.60 ± 0.66	12.40 ± 0.88
Hp. EtAc	100	19.96 ± 0.05	22.26 ± 0.04	24.80 ± 0.07
Hp. EtAc + NLX	100 + 1	11.30 ± 0.03	13.70 ± 0.09	16.60 ± 0.48
Hp. Chf	100	20.42 ± 0.08	25.60 ± 0.05	28.10 ± 0.55
Hp. Chf + NLX	100 + 1	8.53 ± 0.30	9.98 ± 0.59	14.40 ± 0.71

While after 60 minutes, the standard drug tramadol possesses a significant result (*P* < 0.01). Hp. Chf and Hp. EtAc fractions displayed dose-dependent result causing 61.01 ± 0.45 (*P* < 0.001) and 38.45 ± 0.50 (*P* < 0.01) analgesic effect at the dose of 100 mg/kg. Similarly, after 90 minutes, tramadol again exhibited a significant result (*P* < 0.01), while tested fraction Hp. EtAc has 33.06 ± 0.33 (*P* < 0.01)% potential. Likewise, tested fraction Hp. Chf at 100 mg/kg body weight exhibited dose-dependent results as that *f* the standard drug causing 48.75 ± 0.56 (*P* < 0.001)% inhibitions.

## Data Availability

Data will be available from the corresponding author upon request.

## Abbreviations

AA: Arachidonic acid
Aq: Aqueous
Bt: Butanol
Cr: Crude extract
Chf: Chloroform
COX: Cyclooxygenase
EtAc: Ethyl acetate
GC-MS: Gas chromatography-mass spectroscopy
GIT: Gastrointestinal tract
Hp: *Habenaria plantaginea*
IL-6: Interleukine-6
KH2PO4: Potassium dihydrogen phosphate
K2HPO4: Dipotassium hydrogen phosphate
LOX: Lipoxygenase
NSAIDs: Nonsteroidal anti-inflammatory drugs
PGH2: Prostaglandin H2
ROS: Reactive oxygen species
SEM: Standard error means
TMPD: N,N,N-Tetramethyl-p-phenylenediamine dihydrochloride
TNF-*α*: Tumor necrosis factor-alpha.
